# Nasal Intermittent Positive Pressure Ventilation and Bronchopulmonary Dysplasia Among Very Preterm Infants Never Intubated During the First Neonatal Admission: A Multicenter Cohort Study

**DOI:** 10.3389/fped.2022.896331

**Published:** 2022-04-27

**Authors:** Alejandro Avila-Alvarez, Fermín García-Muñoz Rodrigo, Gonzalo Solís-García, Sonia Pertega-Diaz, Manuel Sánchez Luna, Martin Iriondo-Sanz, Dolores Elorza Fernandez, Carlos Zozaya

**Affiliations:** ^1^Neonatal Unit, Department of Pediatrics, Complejo Hospitalario Universitario A Coruña, A Coruña, Spain; ^2^A Coruña Biomedical Research Institute (INIBIC), A Coruña, Spain; ^3^Division of Neonatology, Complejo Hospitalario Universitario Insular - Materno Infantil, Las Palmas de Gran Canaria, Spain; ^4^Division of Neonatology, Hospital for Sick Children, University of Toronto, Toronto, ON, Canada; ^5^Research Support Unit, Complejo Hospitalario Universitario A Coruña, A Coruña, Spain; ^6^Division of Neonatology, Hospital General Universitario Gregorio Marañón, Madrid, Spain; ^7^Division of Neonatology, Hospital Sant Joan de Déu, BCNatal, Barcelona University, Barcelona, Spain; ^8^Division of Neonatology, Hospital Universitario La Paz, Madrid, Spain

**Keywords:** very preterm infants, bronchopulmonary dysplasia, non-invasive ventilation, preterm outcomes, nasal intermittent positive pressure ventilation

## Abstract

**Introduction:**

While non-invasive positive-pressure ventilation (NIPPV) is increasingly used as a mode of respiratory support for preterm infants, it remains unclear whether this technique translates into improved respiratory outcomes. We assessed the association between NIPPV use and bronchopulmonary dysplasia (BPD)-free survival in never intubated very preterm infants.

**Methods:**

This multicenter cohort study analyzed data from the Spanish Neonatal Network SEN1500 corresponding to preterm infants born at <32 weeks gestational age and <1,500 g and not intubated during first admission. The exposure of interest was use of NIPPV at any time and the main study outcome was survival without moderate-to-severe BPD. Analyses were performed both by patients and by units. Primary and secondary outcomes were compared using multilevel logistic-regression models. The standardized observed-to-expected (O/E) ratio was calculated to classify units by NIPPV utilization and outcome rates were compared among groups.

**Results:**

Of the 6,735 infants included, 1,776 (26.4%) received NIPPV during admission and 6,441 (95.6%) survived without moderate-to-severe BPD. After adjusting for confounding variables, NIPPV was not associated with survival without moderate-to-severe BPD (OR 0.84; 95%CI 0.62–1.14). A higher incidence of moderate-to-severe BPD-free survival was observed in high- vs. very low-utilization units, but no consistent association was observed between O/E ratio and either primary or secondary outcomes.

**Conclusion:**

NIPPV use did not appear to decisively influence the incidence of survival without moderate-to-severe BPD in patients managed exclusively with non-invasive ventilation.

## Introduction

Avoidance of invasive mechanical ventilation (IMV) is among the highest priorities of modern neonatal care and, globally, infants are managed less-invasively now than decades ago (1, 2). The mainstay of this non-invasive approach is prioritization of initial stabilization with continuous positive airway pressure (CPAP) rather than prophylactic intubation. However, this strategy still fails in a significant proportion of infants and emerging evidence suggests that its incorporation into clinical practice has not significantly improved rates of bronchopulmonary dysplasia (BPD) (2–6).

Efforts to reduce CPAP failure and potentially decrease the incidence of BPD prompted the incorporation of other modes of non-invasive ventilation (NIV). Nasal intermittent positive-pressure ventilation (NIPPV) is a type of NIV that combines intermittent ventilator inflations with CPAP throughout the respiratory cycle. NIPPV can be provided by conventional ventilators or bi-level CPAP devices, and the intermittent inflations may or may not be synchronized with the infant’s spontaneous breathing (7). This technique has become popular in some countries and is widely used with different indications (8–11).

Available evidence suggests that the incidence of respiratory failure and the need for intubation is reduced significantly by NIPPV vs. CPAP when used for primary respiratory support (12–15). Whether this translates into improved in-hospital respiratory outcomes is less clear, since the majority of individual studies and meta-analyses report little or no effect on BPD rates (13, 16). Most studies comparing CPAP and NIPPV include infants who were intubated at some point during neonatal admission (before, after, or in between periods of NIV). Those periods of IMV may have modified the risk of chronic respiratory morbidity. However, in current neonatal medicine many preterm babies are stabilized with NIV and are never intubated, or are only briefly intubated for surfactant administration (1).

The present study investigated the association between the use of NIPPV and BPD-free survival in very preterm infants managed non-invasively. We hypothesized that the use of NIPPV would increase the probability of BPD-free survival.

## Materials and Methods

### Study Design and Population

This multicenter cohort study is a retrospective analysis of data collected prospectively from infants who were born with a birth weight <1,500 g and/or at <32 weeks gestational age (GA), and were admitted to centers of the SEN1500 network. For this study, we selected patients born between 23^0/7^ and 31^6/7^ weeks GA who were managed exclusively with NIV. Outborn patients, infants who died in the delivery room (DR), and those with major congenital anomalies, as well as infants from units with intermittent data input, were excluded from the analysis, as were patients who did not receive any type of respiratory support. The study period was from January 2010 to December 2019.

### Outcome Variables and Definitions

The exposure of interest was NIPPV (synchronized and non-synchronized, bilevel and ventilator-delivered) at any time during the neonatal intensive care unit (NICU) stay. Infants were classified into two groups: the study group comprised patients who received NIPPV at any time during admission, while the control group consisted of patients managed only with CPAP and/or high flow nasal cannula (HFNC).

The primary outcome was survival without moderate-to-severe BPD until discharge from hospital. Secondary outcomes were survival without BPD, survival, BPD, gastrointestinal perforation, necrotizing enterocolitis (NEC), patent ductus arteriosus (PDA), pneumothorax, intraventricular hemorrhage (IVH), and home oxygen. BPD was defined as the need for supplementary oxygen for at least 28 days and classified as moderate or severe depending on oxygen requirements and ventilator support at 36 weeks postmenstrual age (17, 18).

### Statistical Analysis

Data are presented as the mean ± standard deviation or n (%). Basal and demographic characteristics, as well as interventions and predefined outcomes, were compared between the study and control group. For univariate analyses the Student *t*-test and Mann-Whitney *U* test were used for continuous variables and the Chi-squared test or Fisher exact test for categorical variables, as appropriate.

The odds ratio (OR) for the primary and secondary outcomes were then compared between groups by two different multilevel logistic-regression models, one adjusted only for GA (model 1) and another adjusted for pre-defined confounding variables: GA, sex, small for GA (SGA), prenatal steroids, multiple gestation, and surfactant (model 2). A multilevel approach, including hospital identifier as a random effect, was considered to account for clustering of patients within hospitals. The adjusted OR with corresponding 95% confidence interval (CI) were calculated.

In addition to the analysis by individual patients, an analysis by units was performed. To this end, unadjusted rates of NIPPV use were calculated per unit (i.e., proportion of patients that received NIPPV at some point). Given that units assist children with different demographic and perinatal characteristics, and clinical management also differs between units, an expected rate of NIPPV utilization was calculated for each hospital by a logistic regression analysis adjusting for confounding variables. The results from this model were used to calculate the probability of receiving NIPPV for each newborn. The expected rate of NIPPV utilization for each hospital was then computed by averaging the predicted probability for each individual newborn within that hospital. Subsequently, for each unit, the standardized observed-to-expected ratio (O/E) was calculated. Ratios >1 indicate higher-than-average use, while ratios <1 indicate hospitals with lower NIPPV use.

NICUs were classified as very low-, low-, medium- or high-utilization units based on the quartiles of the O/E ratio of NIPPV use, and outcome rates were compared among these groups. To further analyze the relationship between the standardized O/E ratio of NIPPV use and the different outcomes, we applied a flexible simple regression approach. Each of the outcomes was considered as the dependent variable, including the O/E ratio as a continuous independent covariate. To avoid the linearity assumption, the ratio was modeled using cubic b-splines with three degrees of freedom. Finally, a multilevel logistic regression model was established, including the quartiles of the O/E ratio of NIPPV use as an independent factor, adjusting for the same pre-defined confounding variables.

*P*-values < 0.05 were considered statistically significant. Statistical analysis was performed with Stata 13.1 (StataCorp, College Station, TX, United States) and R 4.0 statistical software with the libraries *splines* and *lme4* added.

## Results

**FIGURE 1:**
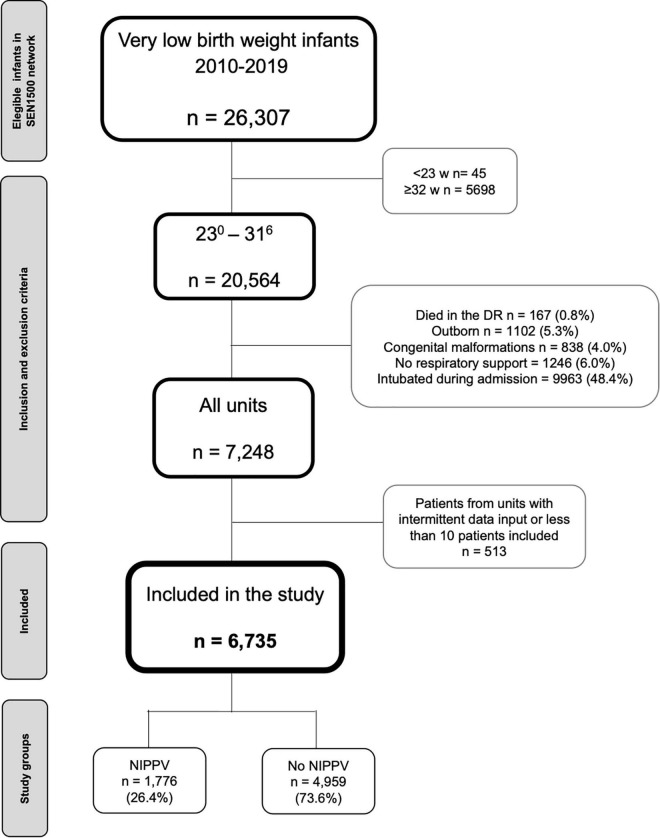
Flow chart depicting the recruitment of the cohort. DR, delivery room; NIPPV, non-invasive positive pressure ventilation.

A total of 26,307 VLBW infants were admitted to participating units during the study period. Of these, 6,735 infants were ultimately included in the analysis after applying exclusion criteria (shown in Figure 1). The mean GA and birthweight of the study sample were 29.6 ± 1.5 weeks and 1,175.8 ± 222.9 g, respectively.

In total, 1,776 patients (26.4%) received NIPPV during NICU admission and were designated as the NIPPV group. The remaining patients (*n* = 4,959, 73.6%) were assigned to the control group. Infants in the NIPPV group had a lower GA and birthweight, and more frequently received supplemental oxygen, CPAP, or NIPPV in DR, and surfactant, HFNC, and steroids during admission (Table 1). There were no other significant differences between groups.

**TABLE 1:** Demographic and perinatal characteristics and interventions performed in the delivery room and during the NICU admission in the two study groups.

	NIPPV *n* = 1,776	No NIPPV *n* = 4,959	*p*-value
Gestational age (weeks)	29.1 ± 1.6	29.7 ± 1.4	**<0.001**
Distribution by gestational age			**<0.001**
23^0^–25^6^	64 (3.6)	41 (0.8)	
26^0^–28^6^	664 (37.4)	1,270 (25.6)	
29^0^–31^6^	1,048 (59.0)	3,648 (73.6)	
Birth weight (g)	1,127.9 ± 235.0	1,192.9 ± 215.9	**<0.001**
Female	871 (49.1)	2,543 (51.3)	0.106
Cesarean section	1,259 (70.9)	3,511 (70.8)	0.944
Chorioamnionitis	346 (19.5)	820 (16.5)	**0.005**
CRIB score	**1.7 (1.9)**	**1.3 (1.4)**	**<0.001**
Maternal arterial hypertension	356 (20.0)	1,037 (20.9)	0.439
Multiple birth	591 (33.3)	1,727 (34.8)	0.239
IVF	424 (23.9)	1,021 (20.6)	0.004
SGA	232 (13.1)	661 (13.3)	0.776
Antenatal steroids (at least one dose)	1,713 (96.7)	4,720 (95.6)	0.051
Supplemental oxygen in the DR	1,307 (73.6)	3,163 (63.8)	**<0.001**
CPAP in the DR	1,463 (82.4)	3,688 (74.4)	**<0.001**
NIPPV in the DR	1,016 (57.2)	2,370 (47.8)	**<0.001**
Surfactant (any time)	524 (29.5)	736 (14.8)	**<0.001**
CPAP during admission	1,705 (96.0)	4,884 (98.5)	**<0.001**
HFNC during admission	772 (43.5)	1,481 (29.9)	**<0.001**
Steroids for BPD	44 (2.5)	67 (1.4)	**0.001**
Steroids for BPD, day of life	31.8 ± 19.7	32.6 ± 15.3	0.824

*BPD, bronchopulmonary dysplasia; CPAP, continuous positive airway pressure; CRIB, clinical risk index for babies; DR, delivery room; HFNC, high flow nasal cannula; IVF, in vitro fertilization; NIPPV, non-invasive positive pressure ventilation; SGA, small for gestational age. Data expressed as the mean ± standard deviation for quantitative variables and n (%) for qualitative variables. Bold indicates statistical significance.*

In the unadjusted analysis the NIPPV group showed a lower frequency of survival without moderate-to-severe BPD (94.5 vs. 96.0%; *p* < 0.001) and BPD-free survival (81.9 vs. 86.4%; *p* < 0.001) than the control group. Moreover, the incidence of BPD, moderate-to-severe BPD, severe IVH, medically treated PDA, and domiciliary oxygen were higher in the NIPPV than the control group. No significant differences in other secondary outcomes were observed (Table 2).

**TABLE 2:** Primary and secondary outcomes according to the use of non-invasive positive pressure ventilation (NIPPV).

	No NIPPV		NIPPV		Unadjusted analysis		Adjusted analysis^a^		Adjusted analysis^b^	
					
	n	%	n	%	*p*	OR (95% CI)	*p*	OR (95% CI)	*p*	OR (95% CI)
**Primary outcome:**										
Moderate-to-severe BPD-free survival	4,762	96.0%	1,679	94.5%	<0.001	0.48 (0.36–0.65)	0.013	0.68 (0.51–0.92)	0.263	0.84 (0.62–1.14)
**Secondary outcomes:**										
BPD-free survival	4,286	86.4%	1,454	81.9%	<0.001	0.42 (0.35–0.50)	<0.001	0.67 (0.55–0.81)	0.049	0.81 (0.66–0.99)
Survival	4,933	99.5%	1,769	99.6%	0.500	1.33 (0.58–3.07)	0.077	2.17 (0.92–5.12)	0.046	2.40 (1.01–5.68)
BPD	636	12.9%	306	17.3%	<0.001	2.41 (2.01–2.90)	<0.001	1.50 (1.23–1.83)	0.047	1.23 (1.00–1.52)
Moderate-to-severe BPD	172	3.5%	90	5.1%	<0.001	2.28 (1.68–3.10)	<0.001	1.66 (1.21–2.27)	0.072	1.34 (0.97–1.85)
Pneumothorax	30	0.6%	19	1.1%	0.086	1.70 (0.93–3.11)	0.075	1.75 (0.95–3.23)	0.514	1.23 (0.66–2.31)
Discharged home on oxygen	65	1.3%	44	2.5%	<0.001	3.03 (1.92–4.78)	0.001	2.15 (1.35–3.44)	0.009	1.90 (1.18–3.07)
Medically treated PDA	442	8.9%	242	13.6%	<0.001	1.94 (1.59–2.36)	<0.001	1.46 (1.19–1.79)	0.092	1.20 (0.97–1.48)
SIP	20	0.4%	5	0.3%	0.474	0.69 (0.25–1.90)	0.203	0.52 (0.19–1.43)	0.127	0.45 (0.16–1.25)
Necrotizing enterocolitis	142	2.9%	63	3.5%	0.213	1.24 (0.88–1.75)	0.547	1.11 (0.79–1.58)	0.812	1.04 (0.73–1.49)
Intraventricular hemorrhage > II	77	1.6%	45	2.5%	<0.001	2.69 (1.73–4.19)	0.002	2.05 (1.30–3.23)	0.009	1.85 (1.17–2.94)

*Unadjusted and adjusted odds ratios and 95% confidence interval from multilevel logistic regression analysis.*

*CI, confidence interval; OR, odds ratio; NIPPV, non-invasive positive pressure ventilation; SIP, Spontaneous intestinal perforation.*

*^a^Adjusted for gestational age.*

*^b^Adjusted for gestational age, sex, small for gestational age, prenatal steroids, multiple gestation, chorioamnionitis, and surfactant.*

After adjusting for GA (model 1) survival without moderate-to-severe BPD remained inversely associated with NIPPV use (OR 0.68; 95%CI 0.51–0.92). However, after adjusting for prespecified confounding variables (model 2) this association disappeared (OR 0.84; 95%CI 0.62–1.14). Significant associations persisted for other secondary outcomes, such as BPD-free survival, home oxygen, and severe IVH (Table 2). These results remained unchanged when focusing in the specific population of infants under 30 weeks GA (Supplementary Tables 1, 2).

**FIGURE 2:**
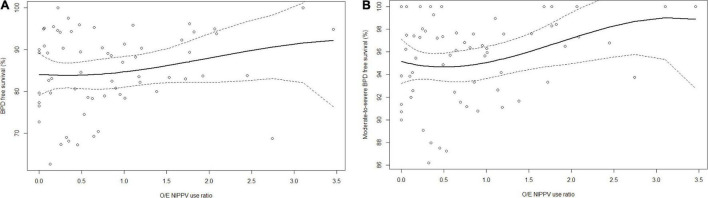
Relationships between O/E ratio of NIPPV use and **(A)** BPD and **(B)** survival without moderate-to-severe BPD. Solid line represents fitted flexible regression curve using a cubic b-splines basis with three degrees of freedom. Dashed lines represent 95% confidence intervals.

In the analysis by units, mean observed NIPPV use was 27.7 ± 20.4% (range, 0–89.7%). After applying a logistic regression model and adjusting for potential confounding variables, expected NIPPV rates by unit ranged from 21.8 to 37.9%. Accordingly, the mean O/E ratio was 0.8 ± 0.8 (range, 0–3.5). We observed no significant and consistent association between O/E ratio by units and either primary or secondary outcomes, except for a higher incidence of survival without moderate-to-severe BPD in high-utilization vs. very-low-utilization units (shown in Table 3 and Figure 2).

**TABLE 3:** Primary and secondary outcomes according to the hospital rate of non-invasive positive pressure ventilation (NIPPV).

	Quartiles of O/E ratio of NIPPV use			

	Very low-utilization NICUs (O/E ratio ≤ 0.22)	Low-utilization NICUs (O/E ratio 0.23–0.63)	Medium-utilization NICUs (O/E ratio 0.64–1.18)	High-utilization NICUs (O/E ratio ≥ 1.19)
No. units	16	16	16	16
No. patients (%)	1,404 (20.8%)	1,437 (21.3%)	1,713 (25.4%)	2,181 (32.4%)
Observed NIPPV use rate (min-max)	0–5.4%	5.6–18.9%	16.9–29.1%	30.7–89.7%

	**OR (95% CI)** ^a^	**OR (95% CI)** ^a^	**OR (95% CI)** ^a^	**OR (95% CI)** ^a^

**Primary outcome:**				
Moderate-to-severe BPD free survival	1	1.01 (0.55–1.83)	1.14 (0.63–2.05)	2.13 (1.15–3.94)
**Secondary outcomes:**				
BPD-free survival	1	0.95 (0.50–1.83)	1.21 (0.64–2.30)	1.76 (0.92–3.35)
Survival	1	1.06 (0.40–2.82)	1.35 (0.51–3.57)	2.02 (0.72–5.62)
BPD	1	1.06 (0.54–2.07)	0.83 (0.43–1.61)	0.57 (0.29–1.10)
Moderate-to-severe BPD	1	0.98 (0.49–1.95)	0.90 (0.46–1.76)	0.45 (0.22–0.91)
Pneumothorax	1	1.77 (0.64–4.92)	1.63 (0.58–4.60)	1.68 (0.64–4.45)
Discharged home on oxygen	1	0.89 (0.24–3.30)	0.91 (0.26–3.23)	0.31 (0.08–1.25)
Medically treated PDA	1	0.92 (0.54–1.55)	0.97 (0.58–1.64)	0.70 (0.41–1.18)
Spontaneous intestinal perforation	1	1.04 (0.31–3.46)	0.72 (0.20–2.54)	0.54 (0.14–1.99)
Necrotizing enterocolitis	1	0.88 (0.67–1.15)	0.80 (0.61–1.04)	0.83 (0.63–1.10)
Intraventricular hemorrhage > II	1	1.94 (0.65–5.75)	1.41 (0.47–4.21)	1.53 (0.52–4.50)

*Data represent the adjusted odds ratios and 95% confidence interval from multilevel logistic regression analysis.*

*NICU, neonatal intensive care unit; CI, confidence interval; O/E, observed rate to expected ratio of NIPPV use; OR, odds ratio; NIPPV, non-invasive positive pressure ventilation.*

*^a^Adjusted for gestational age, sex, small for gestational age, prenatal steroids, multiple gestation, chorioamnionitis, and surfactant.*

## Discussion

In this large, retrospective, multicenter, national cohort study we used patient and unit-based approaches to explore the relationship between NIPPV use and BPD among preterm infants that were successfully managed without IMV throughout admission. Our findings show that the use of NIPPV does not appear to decisively improve the probability of survival without BPD.

Non-invasive positive-pressure ventilation is widely used in adults and children with respiratory insufficiency (19, 20). It was first used in neonatology in the 1980s, but the last decade has seen renewed interest in NIPPV in an effort to reduce the frequency of CPAP failure. A survey of practice in 2008 found that NIPPV was used by 44 of 91 (48%) English neonatal units, with considerable variability (11) and we recently reported increasing use of NIPPV in very preterm infants in Spain (3).

Differences in mode and device terminology, as well as study designs, complicate the interpretation of published evidence on the relationship between NIPPV and BPD. The most relevant data come from trials comparing the efficacy of NIPPV vs. the standard of treatment (i.e., CPAP) in heterogeneous preterm infant populations. The largest randomized controlled trial (RCT) was published in 2013 by Kirpalani et al. (21). The authors randomized 1,009 infants <1,000 g and <30 weeks GA to either NIPPV or CPAP whenever NIV was going to be used for the first time. In line with our findings, NIPPV was not associated with a significant reduction in death or BPD.

Since the first trials comparing CPAP and NIPPV and showing promising results (22, 23), some 15 RCTs and several observational studies have specifically evaluated NIPPV as primary respiratory support. While some of these studies reported short-term benefits associated with NIPPV (mainly a reduction in the need for IMV), few differences were observed in the rates of BPD or other relevant outcomes (16, 22–27).

A Cochrane review that included many of the aforementioned RCTs found that, compared with CPAP as a primary mode, NIPPV was associated with a reduced need for intubation, with a relative risk (RR) of 0.78 (95%CI 0.64–0.94) (13), but observed no reduction in BPD risk (RR 0.78, 95%CI 0.58–1.06). As in the present study, that meta-analysis included NIPPV delivered by a ventilator or by bilevel devices, as well as synchronized and non-synchronized modes.

The aforementioned Cochrane review was followed by at least three other meta-analyses. Ekhaguere et al. pooled data from 16 trials and reported findings similar to those of the Cochrane review (14). More recently, a comprehensive network meta-analysis compared the efficacy of four different non-invasive respiratory support modes used as the primary method in preterm infants (12). The authors reported that NIPPV was more effective than CPAP in decreasing the requirement for IMV (RR 0.60; CI 95% 0.44–0.77) and resulted in a slightly lower incidence of BPD or mortality (RR 0.74; CI 95% 0.52–0.98).

The most recent meta-analysis is that of Rüegger et al. which analyzed 18 trials with a total of 1,900 infants, and included data from 8 newly published trials not included in the Cochrane study. Pooled data demonstrated a 37% relative reduction in the risk of respiratory failure and a 28% reduction in BPD at 36 weeks, with no differences in mortality. However, this difference in BPD risk was fully attributable to the studies using ventilator-generating synchronized systems (28).

All these trials included infants that received IMV at some point during their clinical course. Hence, we speculate that the conclusions of those studies may not be generalizable to intubation-naïve infants. To the best of our knowledge, no RCTs have focused specifically on the subset of infants managed only with NIV and never intubated, which constitutes an increasingly common profile in neonatal units (3).

Given the overall uncertainty surrounding published findings on the long-term efficacy of NIPPV, European consensus guidelines stated that there is insufficient evidence to recommend NIPPV as a primary mode of respiratory support for preterm infants (1). Notably, the mechanism of action of NIPPV itself is not yet completely understood and there is little information available to help clinicians optimize NIPPV settings. Some of the benefits seen in adult and children populations (19, 20) may not be replicated in neonatal patients due to anatomical differences, distinct pathophysiological pathways, or the use of different interfaces.

The most likely mechanism accounting for the greater reduction in BPD observed with NIPPV vs. CPAP is avoidance of IMV. However, the pathogenesis of BPD is complex and a single intervention is unlikely to significantly alter its incidence. Our study population did not include patients who failed CPAP and required intubation during admission, and even though we adjusted for the main confounding variables, this may have biased our sample selection by underestimating the BPD rate in the CPAP group. Moreover, infants in the NIPPV group were significantly smaller and probably sicker, which might translate into higher basal risk for BPD. Encouragingly, we observed no significant differences in the incidence of previously reported NIPPV-associated complications, such as gastrointestinal perforation (9). The observed association between NIPPV and both severe intraventricular hemorrhage and domiciliary oxygen in the multivariate analysis are worrisome findings that warrant further study.

The present study has some limitations. The database used did not record data on NIPPV indication, timing, duration, the devices used, synchronization, interfaces, or settings, nor were these parameters standardized in the participating centers. The combination of different devices and techniques in our series could have contributed to the apparent absence of a beneficial effect of NIPPV. However, a previous meta-analysis (13) and a large RCT (21) both used a similarly broad definition of NIPPV technique and indications, an approach that the respective authors considered pragmatic. Limitations inherent to population-based cohorts, such as inaccuracy in some data, cannot be excluded in this analysis. Strengths of our study include the large size of the sample of non-invasively managed infants, its multicenter nature, and the detailed evaluation of multiple clinical outcomes.

In conclusion, in this large, national-based cohort the use of NIPPV appeared not to decisively influence the incidence of survival without moderate-to-severe BPD in patients managed exclusively with NIV. Differences in the basal risk for BPD between groups and the better outcomes in high NIPPV-utilization units may show that NIPPV could in fact be protective. Uncertainty thus remains as to NIPPV efficacy in the context of longer-term outcomes. In our opinion, more data on the indications, settings, and physiological basis for NIPPV are needed before this approach can be considered as standard of treatment.

## Data Availability Statement

The raw data supporting the conclusions of this article will be made available by the authors, without undue reservation.

## Ethics Statement

The studies involving human participants were reviewed and approved by A Coruña-Ferrol Research Ethics Committee (Ref 2017/360, first author institution). Primary data collection was approved by the local ethics research committees of the participating centers when they joined the SEN1500 Network. This study protocol has no specific ethical approval as it only gathers anonymized data. Written informed consent to participate in this study was provided by the participants’ legal guardian/next of kin.

## Author Contributions

AA-A developed the research idea. AA-A, FG-MR, and CZ designed the protocol and requested the data. AA-A and SP-D analyzed the data. AA-A wrote the initial draft of the manuscript, which was critically revised by FG-MR, CZ, MSL, SP-D, DE, GS-G, and MI-S. All authors contributed to the article and approved the submitted version.

## Conflict of Interest

The authors declare that the research was conducted in the absence of any commercial or financial relationships that could be construed as a potential conflict of interest.

## Publisher’s Note

All claims expressed in this article are solely those of the authors and do not necessarily represent those of their affiliated organizations, or those of the publisher, the editors and the reviewers. Any product that may be evaluated in this article, or claim that may be made by its manufacturer, is not guaranteed or endorsed by the publisher.
