# Supplementary material for: Nasal Intermittent Positive Pressure Ventilation and Bronchopulmonary Dysplasia Among Very Preterm Infants Never Intubated During the First Neonatal Admission: A Multicenter Cohort Study
Source: Front Pediatr. 2022 Apr 27;10:896331. doi: 10.3389/fped.2022.896331 (PMC9091508; doi:10.3389/fped.2022.896331)
Supplement: Supplementary file 1 [file Data_Sheet_1.docx]

###### Supplementary material.

###### Table S1. Demographic and perinatal characteristics and interventions performed in the delivery room and during the NICU admission in the two study groups. Data limited to infants <30 weeks´ GA and birth weight <1000 g

|  | **NIPPV n = 456** | | | | **No NIPPV n = 771** | | | |  |
| --- | --- | --- | --- | --- | --- | --- | --- | --- | --- |
|  | **Mean** | **SD** | **n** | **%** | **Mean** | **SD** | **n** | **%** | **p** |
| **Gestational age (weeks)** | 27,49 | 1,30 |  |  | 27,98 | 1,23 |  |  | <0.001 |
| **Birth weight (grams)** | 842 | 119 |  |  | 868 | 114 |  |  | <0.001 |
| Female |  |  | 252 | 55,3% |  |  | 448 | 58,1% | 0.331 |
| Cesarean section |  |  | 332 | 72,8% |  |  | 599 | 77,7% | 0.053 |
| **Chorioamnionitis** |  |  | 120 | 26,3% |  |  | 151 | 19,6% | 0.006 |
| Maternal arterial hypertension |  |  | 98 | 21,5% |  |  | 195 | 25,3% | 0.131 |
| Multiple birth |  |  | 118 | 25,9% |  |  | 216 | 28,0% | 0.416 |
| IVF |  |  | 86 | 18,9% |  |  | 162 | 21,0% | 0.364 |
| SGA |  |  | 92 | 20,2% |  |  | 170 | 22,0% | 0.439 |
| Antenatal steroids (at least one dose) |  |  | 449 | 98,5% |  |  | 744 | 96,7% | 0.069 |
| **Supplemental oxygen in the DR** |  |  | 379 | 83,1% |  |  | 566 | 73,4% | <0.001 |
| **CPAP in the DR** |  |  | 401 | 87,9% |  |  | 626 | 81,2% | 0.002 |
| NIPPV in the DR |  |  | 285 | 62,5% |  |  | 442 | 57,3% | 0.075 |
| **Surfactant (any time)** |  |  | 139 | 30,5% |  |  | 166 | 21,5% | <0.001 |
| **CPAP during admission** |  |  | 441 | 96,7% |  |  | 763 | 99,0% | 0.005 |
| **HFNC during admission** |  |  | 257 | 56,4% |  |  | 296 | 38,4% | <0.001 |
| Steroids for BPD |  |  | 22 | 4,8% |  |  | 36 | 4,7% | 0.901 |
| Steroids for BPD, day of life | 35,5 | 20,5 |  |  | 30,1 | 13,5 |  |  | 0.309 |

*BPD, bronchopulmonary dysplasia; CPAP, continuous positive airway pressure; CRIB, clinical risk index for babies; DR, delivery room; HFNC, high flow nasal cannula; IVF, in vitro fertilization; NIPPV, noninvasive positive pressure ventilation; SGA, small for gestational age. Data expressed as the mean ± standard deviation for quantitative variables and n (%) for qualitative variables. Bold indicates statistical significance.*

**Table S2.** Primary and secondary outcomes according to the use of non-invasive positive pressure ventilation (NIPPV). Unadjusted and adjusted odds ratios and 95% confidence interval from multilevel logistic regression analysis. **Data limited to infants <30 weeks´ GA and birth weight <1000 g.**

|  | **Unadjusted analysis** | | | | **Adjusted analysis^a^** | | | | **Adjusted analysis^b^** | | | |
| --- | --- | --- | --- | --- | --- | --- | --- | --- | --- | --- | --- | --- |
|  | **p** | **OR** | **95% CI** | | **p** | **OR** | **95% CI** | | **p** | **OR** | **95% CI** | |
| **Primary outcome:** |  |  |  |  |  |  |  |  |  |  |  |  |
| BPD-free survival | **<0.001** | 0,58 | 0,43 | 0,77 | **0.034** | 0,72 | 0,54 | 0,98 | 0.237 | 0,83 | 0,61 | 1,13 |
| **Secondary outcomes:** |  |  |  |  |  |  |  |  |  |  |  |  |
| Moderate-to-severe BPD free survival | **0.008** | 0,58 | 0,39 | 0,87 | **0.037** | 0,65 | 0,43 | 0,97 | 0.160 | 0,74 | 0,49 | 1,12 |
| Survival | 0.329 | 0,60 | 0,21 | 1,68 | 0.100 | 0,41 | 0,14 | 1,18 | 0.102 | 0,41 | 0,14 | 1,19 |
| BPD | **<0.001** | 1,78 | 1,33 | 2,39 | **0.026** | 1,41 | 1,04 | 1,92 | 0.168 | 1,25 | 0,91 | 1,72 |
| Moderate-to-severe BPD | **0.002** | 2,00 | 1,30 | 3,08 | **0.005** | 1,88 | 1,21 | 2,91 | **0.030** | 1,64 | 1,05 | 2,58 |
| Pneumothorax | 0.440 | 0,42 | 0,05 | 3,78 | 0.383 | 0,37 | 0,04 | 3,43 | 0.300 | 0,31 | 0,03 | 2,86 |
| Discharged home on oxygen | **0.005** | 2,54 | 1,32 | 4,89 | **0.012** | 2,34 | 1,20 | 4,56 | **0.024** | 2,18 | 1,11 | 4,29 |
| Medically treated PDA | **0.004** | 1,59 | 1,16 | 2,17 | 0.081 | 1,33 | 0,96 | 1,84 | 0.251 | 1,21 | 0,87 | 1,69 |
| Spontaneus intestinal perforation | 0.746 | 1,21 | 0,38 | 3,84 | 0.971 | 1,02 | 0,32 | 3,32 | 0.977 | 0,98 | 0,30 | 3,23 |
| Necrotizing enterocolitis | 0.148 | 0,64 | 0,35 | 1,17 | 0.107 | 0,60 | 0,33 | 1,11 | 0.078 | 0,57 | 0,31 | 1,07 |
| Intraventricular hemorrhage >II | **0.004** | 3,26 | 1,47 | 7,24 | **0.031** | 0,61 | 0,46 | 0,82 | 0.052 | 2,29 | 0,99 | 5,27 |

*Abbreviations: CI, confidence interval; OR, odds ratio; NIPPV, noninvasive positive pressure ventilation; SIP, Spontaneous intestinal perforation*

*^a^Adjusted for gestational age. ^b^Adjusted for gestational age, sex, small for gestational age, prenatal steroids, multiple gestation, chorioamnionitis and surfactant.*

###### Hospitals and investigators of the Spanish Neonatal Network SEN1500

###### H. GERMANS TRIAS I PUJOL (Gemma Ginovart); H. CLÍNIC DE BARCELONA (Josep Figueras Aloy); H. U. BASURTO (Alberto Pérez Legorburu); H.G. DE CASTELLÓN (Ramon Aguilera Olmos); H.U. DE SAN CECILIO (Eduardo Narbona López); H. DE LEON (Sandra Terroba); H. CARLOS HAYA (Tomás Sanchez-Tamayo); H.I. LA PAZ (Mª Dolores Elorza Fernandez); H. CLÍNICO SAN CARLOS (Araceli Corredera Sánchez); H. CENTRAL DE ASTURIAS (Belén Fernández Colomer); COMPLEXO HOSPITALARIO PONTEVEDRA (Mª Angeles Martinez Fernandez); H. UNIVERSITARIO MARQUES DE VALDECILLA (Mª Isabel de las Cuevas Terán); H. DONOSTIA (Miguel Ángel Cortajarena Altuna); H.U.I. VIRGEN DEL ROCÍO (Carmen Macias Díaz); H.U. DE CANARIAS (Pedro Fuster Jorge); H. MIGUEL SERVET (Segundo Rite Gracia); H.C.U. LOZANO BLESA (Mª Purificación Ventura Faci); H.U. LA FE (Mª Isabel Izquierdo Macián); H. VIRGEN DE LA SALUD (Ana Belén Escobar Izquierdo); H.C.U. DE SANTIAGO (Mª Luz Couce Pico); H.U. SALAMANCA (Elena Pilar Gutierrez González); H.G.U. GREGORIO MARAÑÓN (S. Zeballos Sarrato); H. SAN PEDRO DE LOGROÑO (Mª Yolanda Ruiz del Prado); H.U.M.I. LAS PALMAS (Lourdes Urquía Martí); H. DE CABUEÑES (Rafael Garcia Mozo); H.U. REINA SOFÍA (Mª Pilar Jaraba Caballero); H.U. DE BURGOS (Cristina de Frutos Martínez); SCIAS-H. BARCELONA (Sílvia Martínez-Nadal); H. SAN JOAN DE DEU (Martin Iriondo); H. DE CRUCES (Amaya Rodriguez Serna); H.G.U. DE ALICANTE (María Gonzalez Santacruz); H. VIRGEN DE LAS NIEVES (Maria Fernanda Moreno Galdo); CORPORACIO PARC TAULÍ (Joan Badia Barnusell); H.U. RIO HORTEGA (Mª Mar Montejo Vicente); H. TXAGORRITXU (Aintzane Euba); H. JUAN XXIII (Mar Albújar); H. DE GETAFE (Irene Cuadrado Perez); INSTITUT DEXEUS; COMPLEJO HOSPITALARIO ALBACETE (Andres Martinez Gutierrez); H. DE LA SANTA CREU I SANT PAU (Gemma Ginovart Galiana); H. SAN PEDRO DE ALCÁNTARA CÁCERES (Mª Jesús López Cuesta); H. SEVERO OCHOA (Mª José Santos Muñoz); ESTRUCTURA ORGANIZADA DE XESTIÓN INTEGRADA DE VIGO. HOSPITAL ALVARO CUNQUEIRO (María Suárez Albo); H. VIRGEN DE LA CONCHA - COMPLEJO ASISTENCIAL DE ZAMORA (Víctor Manuel Marugán Isabel); H. DE JEREZ (María Victoria Ramos Ramos); H. MONTEPRINCIPE (Gerardo Romera Modamio); H. DE ELCHE (Carolina Vizcaíno); H. JUAN RAMÓN JIMÉNEZ (David Mora Navarro); H. VALME DE SEVILLA (Laura Acosta Gordillo); H.U. ARNAU DE VILANOVA DE LLEIDA (Eduard Soler Mir); H. VIRGEN DE LA MACARENA DE SEVILLA (Mercedes Granero Asensio); H.C.U. DE VALENCIA (Dr. Javier Estañ Capell); H.G.U. DE CIUDAD REAL (Miguel Angel García Cabezas); H. DE LA ZARZUELA (D. López Gómez); H. U. DE GIRONA DR. JOSEP TRUETA (Alberto Trujillo Fagundo); H. DE GRANOLLERS (Israel Anquela Sanz); H. PUERTA DEL MAR (Antonio Segado Arenas); H. 12 DE OCTUBRE (Carmen Rosa Pallás Alonso); H.U. NUESTRA SEÑORA DE CANDELARIA (Sabina Romero); H. PUERTA DE HIERRO (Carmen González Armengod); H.U. SANTA LUCIA DE CARTAGENA (Jose María Lloreda García); H. FUENLABRADA (Laura Domingo Comeche); H.G. DE CATALUÑA (Laura Castells Vilella); H. VIRGEN DEL CAMINO DE PAMPLONA (Concepción Goñi Orayen); H.U. QUIRÓN; CLÍNICA CORACHÁN (Mª Dolores Muro Sebastian); COMPLEXO HOSPITALARIO UNIVERSITARIO DE A CORUÑA (Alejandro Avila-Alvarez); HOSPITAL HM PUERTA DEL SUR (Mara Fernandez Diaz); HOSPITAL QUIRÓN SAGRADO CORAZÓN (Elena García Victori); HOSPITAL UNIVERSITARIO FUNDACIÓN JIMENEZ DÍAZ (Tamara Carrizosa Molina).
